# Book Reviews

**Published:** 1826-02

**Authors:** 


### CRITICAL ANALYSIS

OF

### ENGLISH AND FOREIGN LITERATURE,

RELATIVE TO THE VARIOUS BRANCHES OF

### Medical Science.

Quæ laudanda forent, et quæ culpanda, vicissim

Illa, prius, cretâ; mox hæc, carbone, *notamus*.—PERSIUS.

### DIVISION I.

### ENGLISH.

ART. I.—*Researches into the Nature and Treatment of Dropsy in the Brain, Chest, Abdomen, Ovarium, and Skin; in which a more correct and consistent Pathology of these Diseases is attempted to be established, and a new and more successful Method of Treating them recommended and explained.* By JOSEPH AYRE, M. D. Member of the College of Physicians, &c.—8vo. pp. ix. 242. London: Longman and Co. 1825.

AMONG the multitude of works, of all denominations and sizes, that come before us for examination, it is refreshing to meet with one which enables us to lay aside, in a great measure, our critical weapons, and to present it to our readers, without being compelled to notice omissions, defects, or errors, which to pass by in silence might imply, on our parts, either ignorance, neglect, or wilful partiality. The duty of the professional critic is at all times an unpleasant one, independently of the toil and labour which necessarily attend a conscientious discharge of the office; and it is, indeed, gratifying to us when we meet with a performance which enables us to assume the humble, but much more agreeable, task of analysing instead of criticising. We do not hesitate to affirm, that the present work is one of this kind. That the doctrines it contains are absolutely novel, we do not assert; that they may not be insisted upon a little too pertinaciously, and occasionally carried a little too far, we will not undertake to deny: but that the pathological views of Dr. AYRE are sound; that they are, generally speaking, founded on fact; that they are perspicuously detailed, and ably illustrated, we are fully persuaded: and, moreover, we venture to pronounce that this work will hold a permanent rank among the best monographs of modern times, and will shed a beneficial influence on the treatment of this particular class of diseases.

It will be our business, in going through this work *seriatim*, to endeavour to show that this favourable opinion, as well as the slight qualifications with which we have accompanied it, are the result of a careful perusal of the book itself, of which we hope to be able to give so full an account as to lead those who are at a distance from the best sources of information to review and reconsider those doctrines relative to dropsical complaints, which they most probably have imbibed in the course of their medical education: for all systematic writers, in the classification of these affections, evidently display a want of knowledge of their true character, inasmuch as they make dropsy a *disease*, instead of what it most generally is, merely the *effect of some other morbid condition:* but of this more will be said presently.

Dr. Ayre's work has the additional merit of being short. To us especially this is a great boon; and, when we consider the little time professional men are enabled to bestow upon medical literature, it is a merit of no mean kind.

The volume is divided into four chapters: the first treats of the pathology of dropsy; the second describes the various forms of the complaint; the third is devoted to their treatment; and the fourth consists of cases and dissections. A short Preface introduces the work, the principal point of which is to assert the originality of the author's opinions. This is in some degree rendered necessary by the very striking agreement between the doctrines of Dr. Ayre and those advocated in the Dictionnaire abregé des Sciences Medicales, which is dated in the year 1823, and therefore, in justice to our author, we subjoin the following passage:—"Of the general views which I have given of this disease, I must be allowed to observe, that, under the amplest opportunities for verifying them, they have been entertained and acted on by me for a considerable time, and, during the last five years, have formed the substance of an annual lecture to a class of clinical students."

Chapter I. *on the Pathology of Dropsy.*—Dropsy, observes Dr. Ayre, is merely the effect of disease, and its cause must be looked for in that particular condition of the solids by which the effusion is produced. The nature of that morbid state has been variously explained; but the three principal hypotheses that still hold their ground are the following:—1st. That dropsical swellings arise from want of energy in the absorbent vessels; 2dly, from an increased exhalation of the same fluid, from want of tone in the exhalants; and, 3dly, from mechanical obstruction to the free return of blood by the veins, in consequence of schirrus or other disease. These views our author combats in the following manner:—1st. The opinion of a want of tone in the absorbents causing dropsy, is contradicted by the emaciation of the body,—that is, the absorption of the adipose matter still going on; by the readiness with which the absorbents take up mercury in this condition of the system; by the total absence of swelling or accumulation of the fluids in the joints or bursæ mucosæ; and by the ready absorption of ecchymosed spots in anasarcous limbs. Now, we are free to confess that these proofs of the undiminished power of the absorbents are not fully satisfactory to our minds, and would have but little influence upon our opinion, if the facts and reasonings which our author subsequently adduces in support of his views were not more forcible and convincing; for, towards the termination of chronic diseases ending fatally, when emaciation is the most extreme, œdema, evidently from want of power in the absorbents of the extremities, actually does occur almost universally. Nor does it appear a necessary consequence that, because the absorbents of any cavity,—the chest or abdomen, for example,—are defective in power, those of the surface, or those lining the cavities of joints, should be similarly circumstanced: and, with regard to the removal of ecchymosed spots, such occurrences are not always met with; and, when they are, they generally prove that the disease is yielding; so that a supporter of the old theory might be inclined to say that the absorbents had reacquired their lost tone.

Our author's objections to the opinion of dropsy being caused by a want of tone or energy in the exhalants, are much stronger. We conceive this opinion implies, either that the fluid of dropsy may escape *mechanically* from the exhalants, and that such fluid may be identified with another fluid which is *secreted*; or that, if the fluid of dropsy be *secreted*, that secretion may be increased in quantity under a *decrease* in the energy of the secreting vessels. The validity of these objections rests upon the supposed admission that exhalation is identical with secretion.

With regard to dropsy arising from a mechanical obstruction to the blood's return through the veins, by the pressure of parts of a diseased viscus on the vessels passing through it, Dr. Ayre remarks that, in the instance of the liver, which is usually considered to be productive of dropsy from this cause, there are numerous examples of such states of that organ, as well as of the spleen, &c. without any such effusion; and, moreover, when removed by tapping or other means, the dropsy has not returned, though no change has been effected in the structural condition of the liver. Further, if the discharge depended upon a mechanical cause, the water should in every case, be of a uniform fluidity, and the progress of accumulation should likewise be uniform; neither of which circumstances always, or even often, take place.

Our author next proceeds to combat the conclusions deduced from the experiments made by Lower of tying the vena cava of a dog; and we give his observations on the experiment in his own words:—

"The inferences, however, drawn from the experiment are fallacious; for the experimenter, besides overlooking the agency of effects incidental to the operation, has committed the too-common error of reasoning from the lower animals to man; and therefore has assumed, because ascites occurred in the dog, that it would also have happened in the human subject. But there was an effect, here overlooked, which was to be expected to take place in the abdomen of the dog, from the injury done to the surrounding parts by the operation itself, and which would be quite independent of any effect arising out of the experiment. In the human subject, this effect would be the highest form of inflammation, by which coagulable lymph or pus would be poured out upon the surface of the peritoneum. There would, therefore, be inflammation excited in the abdomen of the dog; but, as the lower animals are less easily acted on than man, the inflammation would in this case be in a lower degree. But every degree of inflammation has its particular product. The highest occasions a discharge of pus, whilst the lowest, when seated in a serous membrane, is a larger portion of its proper serous fluid. This, therefore, might be the product of the inflammation which was produced incidentally by the experiment in the abdomen of the dog; and it would be just as reasonable to regard the coagulable lymph in the human subject, which would result from such an experiment, as an effect of the mechanical obstruction, as to consider the fluid effusionin the dog to be so." (P. 7—9.)

Some other observations, all tending to confirm this view, lead to the following conclusion:—

"From these facts, therefore, and others to be presently noticed, it appears to me conclusive, that the dropsical effusion, in whatever part it may be seated, does not arise from any want of tone in the exhalant or absorbent system, or from any mechanical obstruction in the liver or other viscus; but that it proceeds from a morbid action in the cellular or serous tissues; and that this action, as we shall now proceed to show, is allied in its nature to inflammation." (P. 15.)

In support of this view of the subject, our author calls to our recollection, in the first place, that all the phenomena belonging to cases of watery effusion, under other forms of inflammation, are common to those of dropsy: the fluid secreted in consequence of a blister, in some cases of erysipelas, and in pemphigus, afford his illustrations. Again, in some cases of acknowledged inflammation, the fluid varies much in its consistence. Such also is the case in dropsy, sometimes even at different times in the same patient. To these views, however, there are objections. Our author first states, and then answers them. The first objection applies to the total absence of pain in ordinary cases of local or general dropsy. This is accounted for from the little sensibility of the serous membranes under a state of chronic inflammation; so that death shall often ensue from considerable chronic diseases of those parts, without having been denoted by any previous pain during life. The other objection, arising from the sudden effusion of tears or saliva, under certain conditions of the mind or stomach, we consider so frivolous, as hardly to have merited an answer.

In favour of his peculiar opinions, Dr. Ayre next calls in the aid of analogy. That the morbid action producing dropsy is allied to inflammation, he endeavours to show, from the circumstance of its obeying the same laws: for example, it is liable to metastasis, and is, in many instances, remedial of its cause, as is the secretion of a mucous fluid in mucous membranes, or the effusion of coagulable lymph, or of pus, in the highest degree of inflammation. The examples of these remedial effects in dropsical disease, are the cessation of pain in the extremities on their becoming anasarcous, and of an effusion which has begun to take place in a cavity being temporarily suspended, particularly in ovarian dropsy or in hydrocele, where the fluid continues often for many months, or years, without any addition being made to its quantity: as is the case, also, in other inflammations. When a higher excitement is induced, the serous effusion ceases: this is effected by design in the cure of hydrocele, and is often the effect of accident, especially in the ovarian dropsy. An example of this latter kind is given, which, as it occupies but a short space, we shall extract.

"It was the case of a female, who, for the first time, was tapped for an ovarian dropsy, which she had laboured under several years. The discharge through the canula having suddenly stopt when about two-thirds of it was drawn off, the surgeon introduced his probe to remove the obstacle. In doing this, he produced a slight hemorrhage, and some degree of pain; and, for several days succeeding, there was much constitutional irritation and general fever, with considerable risk to the patient's life. After a difficult struggle, she recovered; and with the agreeable result of having the ovarian dropsy so radically cured as to be now, after seventeen years, still free from it." (P. 25, 26.)

In abdominal dropsy, continues our author, the same consequence would ensue, if increased excitement could be safely brought on in the cavity: but, as this is not the case, when it does take place, a fatal result generally ensues. But sometimes the progressive increase of disease may produce the same effect: that is, a patient may be relieved from his dropsy some weeks, or even months, before his death, in consequence of the excitement of the peritoneal covering, which was merely sufficient, in the first instance, to pour out the serous fluid, becoming increased to a degree that is incompatible with the serous secretion.

Here we would ask, is there not some altered state of the lymphatic system also to be taken into the account; or how is the serous fluid disposed of? This, and some other difficulties which occur to us, would induce us to believe that, though inflammation is the fundamental principle which leads to dropsical effusions of the larger cavities especially, there is something peculiar in the nature and character of that inflammation; that the lymphatic system, either primarily or secondarily, acts some part in the progress of the affection; and that it is not merely a difference in the degree of common inflammation which constitutes the essential character of the disease.

The point which Dr. Ayre contends to establish, of the disappearance of a dropsy whilst the original disease continues to run its course unchecked, is illustrated by the detail of two cases. The first is that of an adult, who, in consequence of the indulgence of intemperate habits, became afflicted with ascites, which was ultimately removed through the kidneys: he survived a twelvemonth, without any return of his dropsical symptoms; and, on dissection, the peritoneal covering of the liver and adjoining organs were of a colour almost perfectly white, and of the thickness of chamois leather, and this appearance pervaded the whole abdomen; whilst the viscera generally were so firmly united together, that not even careful dissection could disjoin them. The second case is that of a child, who had ascites as a consequence of mesenteric disease: the water was removed by the use of diuretic medicines, but the child died in a few weeks. The appearances on dissection were similar to those above described.

From these and similar examples, our author assumes—

"That ascites, when proceeding from some visceral disease, (and the principle applies to hydropic effusions from the presence of disease in other cavities,) does so by the gradual extension of the chronic inflammation of the internal cellular or serous tissues of the diseased organ, to its outer external covering; and that, commencing here as from a point, the serous or hydropic inflammation is progressively propagated through the whole of the serous membrane of the cavity. By the disease within the cellular tissue of the diseased viscus increasing, a corresponding increase in these cases will ensue of the disease on the surface of the membrane investing it; until at length a susceptibility to take on a higher action is induced, which only requires any slight occasional cause to establish. Under this condition of an increased excitement in the peritoneal or other serous membrane, coagulable lymph is discharged into its cellular tissue, and a thickening of it takes place; until at length the operation of paracentesis, which in the early stage of the disease was attended with only inconsiderable inconvenience, becomes an adequate cause of a still higher inflammation, which terminates perhaps in suppuration; and, in the post-mortem examination, the serous fluid is found so mixed with coagulable lymph and purulent matter, as to give a whey or milk-white appearance to the mass. The quantity of the serous fluid in these cases is generally small, when compared with what was accumulated in the intervals of former tappings; for the vascular excitement, which occasions the discharge of coagulable lymph, is destructive of that which pours out the serous fluid." (P. 29, 30.)

Another train of arguments, founded upon observations on the quantity of serum contained in the urine, next engages our attention. They have been previously pointed out by Dr. Wells and Dr. Blackall; and to these Dr. Ayre has added other conclusions, the results of his own practice, according to which it appears that, when dropsy is under a subacute form, and of the anasarcous kind, it is idiopathic; and in this state, as well as in the symptomatic form, though in a less degree, the urine is found to contain serum. The quantity of this ingredient denotes, according to our author, the amount of the serous inflammation: it exists, therefore, in the greatest abundance where the effusion into the skin precedes the local dropsy; and, on the contrary, is in smaller quantity where the anasarca succeeds to the other form, since this order in their appearance indicates the existence of a local disease, as a cause both of the local and general affection. It is, therefore, in the subacute and idiopathic forms of dropsy that this state of the urine prevails the most; and it is in this form, also, that the defective action of the kidneys, skin, and bowels, are most considerable. In dropsy following scarlatina, which is idiopathic, there is generally the greatest quantity of serum, and with it the most decided marks of vascular action. But, adds Dr. Ayre, the urine is also found to contain serum in cases of anasarca, when symptomatic of visceral disease; for the disease of the viscus which is the cause of the local inflammation in the serous membrane of the cavity, may produce an adequate degree of that general vascular excitement which gives rise to a discharge in the cellular tissue.

Upon the above passage we are compelled to remark, that it is neither altogether free from confusion nor contradiction; and we are not quite clear that we comprehend the exact tenor of the doctrine intended to be deduced from it. Indeed, our author himself appears to be aware that, practically, many difficulties will occur in the application of the above principle; and more especially we should conceive, where anasarca only is concerned. Dr. Ayre, therefore, in order to render his views as clear as possible, enumerates four distinct conditions of the system by which its occurrence—viz. that of serum in the urine—is regulated: these we insert.

"1. It is in the greatest quantity where, along with a copious and continued effusion, there is a nearly corresponding quickness in the absorption of the serous fluid, and which will occur most commonly when the general excitement precedes, and is a cause of, the local one.

"2. It is consequently, *cæteris paribus*, in a less quantity where the general hydropic excitement of the system succeeds, and is dependent on, the local one.

"3. It is absent, or found only in a minute proportion, in all those cases where the local increased action in the serous membrane is only partially extended to the rest of the system, and where the absorption from the part is inconsiderable; as particularly happens in the encysted kinds: —or,

"4. Where the effusion of the serous fluid has proved remedial of the inflammation producing it: in which case the disease, as it respects the presence of water in a part, may visibly resemble another example, and yet be essentially different from it, by the serous inflammation which produced it in both having ceased, on its occurrence, in one of them." (P. 41, 42.)

Another evidence of the relation existing between dropsy and disease of local excitement, arises from the effects it produces on the general system,—that is to say, emaciation; and which arises from the continued secretion and absorption of the dropsical fluid, creating effects similar to those which arise from the continued discharge of pus from a suppurating surface; and hence this exhausted or cachectical state of the system, which has so often been assigned as the cause of both local and general dropsy, is an effect only.

The remaining portion of this section we pass by, conceiving it to contain some comparisons between suppurating surfaces falling into gangrene, and the same consequence taking place in œdema, which are rather fanciful than just, and against which it would be very easy to adduce powerful objections.

This long and interesting chapter closes with a recapitulation of the pathological conclusions, together with the facts by which they are supported; but which we regret that our limits prevent us from inserting.

Having in the last chapter cleared the ground for his building, Dr. Ayre commences Chapter the IId. by observing that the effusion of the serous fluid in dropsy is to be considered as arising from a local vascular excitement in a serous tissue, which may be either, first, subacute or chronic; secondly, symptomatic or idiopathic; or, thirdly, the serous inflammation may be either local or general. He then proceeds to the consideration of the several forms of the disease, and which are, 1. Hydrocephalus Internus; 2. Hydrothorax; 3. Ascites; 4. Ovarian Dropsy; 5. Anasarca.

The first section treats of Hydrocephalus Internus. If we pass this by without comment, it is not because we undervalue the contents; but because we have had occasion to bring the subject before our readers, and to explain our views regarding it, in a very recent Number.* We therefore proceed to section the second, on Hydrothorax.

The symptoms of this affection, says our author, pertain only remotely to the true disease. The pressure of the water on the vital organs produces a disturbance, from which these symptoms proceed. There is no occasion for us to recapitulate these symptoms; they are well known. None of them, Dr. Ayre remarks, are truly pathognomic of the effusion, but must be taken collectively: besides which, they only prove the effusion, and not the state of previous excitement which has caused it, and which may still be continuing to operate. Hydrothorax may be either idiopathic or symptomatic, and proceed from a local or general cause; but the nature of the inflammation is the same in both. This is a disease not capable of being divided into stages; but, as is the case in hydrocephalus, there is also in hydrothorax a similar degree of serous inflammation, and our object should be to remove or prevent this state. The most usual mode by which this state is induced in serous membranes, is by the extension of chronic inflammation existing in the diseased organ extending to them, and not by mere consent of parts.

The particular diseases within the chest, tending to produce a serous effusion, are next hinted at; and we are told that, in many cases, the danger of the organic disease is inconsiderable, excepting as the cause of this consecutive complaint (effusion); whilst, in others, the dropsy is the sequel of a disease essentially fatal; and to distinguish between these states, is a desideratum in pathology. Our author here takes occasion to lament the little light that post-mortem examinations have shed upon the true relation existing between the diseases of the several viscera and the serous effusions that take place in their cavities: hence, when a disease of the heart is found, such as ossification of its valves, this is usually considered as a mechanical cause of the effusion; and, as this state cannot be remedied, it is thought no means are available for the prevention of the effusion. But, continues Dr. Ayre, the means sometimes successfully used for the removal of the water, now and then have the effect of removing the chronic excitement producing the effusion; and radical cures are occasionally and unexpectedly occurring, from treatment which is supposed to be exclusively suited to the removal of the water.

When effusion takes place in the chest as a consequence of

---

* See Critical Analysis of an "Essay on the Nature, &c. of Water in the Brain, by W. SHEARMAN, M.D." Number for December.

inflammation, it is often looked upon as the result of debility arising from large depletion. This opinion our author combats, first, from considering that such effusion does not take place frequently for some weeks, or even months, after the bleeding was employed; secondly, that, although the debility is general, the effusion is local, and precisely in that cavity which has been the seat of previous disease. From hence he concludes, that the real truth is that, in such cases, the bleeding has not been carried far enough, or has been used too late; or that, during convalescence, sufficient care has not been taken to avoid those causes which tend to keep up or increase the force of the local or general circulation, whence a lower grade of inflammation is left behind in the chest, which produces the subsequent evil. In such patients there will often be found, upon close examination, a permanent difficulty in breathing whilst at rest, or an obstruction to the full expansion of the chest, which circumstances are irreconcileable with the assumed debility as a cause; though our author admits that the effusion into the chest will sometimes occur without any indications of its approach.

Upon these last statements of our author, we are disposed to pause. We conceive that they cannot be admitted in their full extent, and that, without some qualification, they may lead to an unnecessary and uncalled-for activity in the use of the lancet; which is, perhaps, rather too much the error of the present day, upon every slight occasion. Such effusions as Dr. Ayre alludes to are frequently sudden, instead of requiring months to come on. Again, though the debility be general, still how can effusion be expected but in the cavity where the previous disease has existed? or how does he prove that the mischief arises from a lower grade of inflammation being left? or in what does that expression essentially differ from the disease not having, in fact, been cured at all?

But to return to the causes of hydrothorax: there is one, observes Dr. Ayre, which has a much more important influence in producing or predisposing to it, than is generally supposed;—that is, a congestive state of the circulation, brought on sometimes by indulgence in eating, and taking too little exercise. In this state, any slight cause, further disturbing the balance of the circulation, may lead to the serous effusion. Our readers will probably recollect some cases in Dr. Venables' late publication, which go very far in confirming the views and positions of our author.

Section third treats of Ascites.—Abstractedly from its cause, water in the abdomen, says our author, is of little importance; and in this it differs both from hydrothorax and water in the head, where the effusion forms a most important feature of the disease: it is, therefore, to the remote causes that the attention of the practitioner should be directed. This remote cause may be either symptomatic or idiopathic, local or general. When the disease is symptomatic, some viscus is diseased: but then it is necessary that the disease should be making progress; for, if inflammation be not present, mere bulk is not capable of producing this effect. Examples of this truth are daily offered to us in the liver and spleen, as well as in the glands of the mesentery. To produce the serous discharge, it is, however, necessary that the peritoneal coat should participate in the disease, and the rapidity of the accumulation will have reference to the extent or intensity of the excitement. After tapping, it is well known that the effusion takes place more rapidly. This our author accounts for in consequence of a renewal of the inflammation by the operation.

In some instances the water may be absorbed, and the patient be cured as far as regards the dropsy, whilst the serous inflammation has only yielded to a more active grade of disease; so that coagulable lymph is poured out, the peritoneal surfaces become agglutinated, and a fresh and more formidable disease is superinduced.

When ascites is idiopathic, it may proceed from any of the common causes of inflammation. The most frequent of these is cold, which may act either locally or generally: in the latter case, there is generally anasarca, and the progress is rapid; there is fever, and thirst; the blood is cupped, and the urine loaded with serum. The subjects of this form of disease are robust men, and stout full-habited females. When the watery effusion is in large quantity, it may act as a source of further disease on the peritoneal membrane. One of the most obvious evidences of the effects of this pressure, is the entire absorption of the fat from the parietes of the abdomen. Neither do the viscera of the abdomen themselves always escape; though it must be observed that Dr. Ayre, strangely enough, illustrates this remark by a case of hydrothorax, which, though undeniably true, is not quite an instance in point; since it is obvious that the bony parietes of the chest make it impossible to compare one kind of effusion with the other.

The fourth section, on Ovarian Dropsy, we pass by altogether: it is short, and contains nothing deserving of particular comment.

The subject of Anasarca is discussed in section the fifth, and the same principles are applied to all forms of this affection. It is said by our author to consist of a serous inflammation in the cellular tissue of the body, with a serous effusion as its result. We find that this effusion, like those above mentioned, may be either symptomatic or idiopathic, local or general, and the disease derives all its importance from its remote cause.

The idiopathic forms are first mentioned: that is, anasarca arising from cold, from scarlatina, from cold locally applied, in which the effused fluid is of a gelatinous nature, bearing some resemblance to the phlegmasia dolens, or white leg, of the puerperal state, to which Dr. Ayre believes it to be closely allied. Œdema of the feet is sometimes symptomatic of chylopoietic disease, especially in chlorotic females; but most frequently it attends upon visceral diseases, beginning in the lower extremities, but rarely attended with those strong signs of local excitement so obvious in idiopathic anasarca. Its occurrence towards the close of various chronic diseases of a fatal nature, has been referred to pressure made by the water on the iliac veins; but, says Dr. Ayre, in pregnancy, the uterine pressure produces a considerable swelling of the crural veins, without any serous effusion resulting from it. But here we beg to observe that, in this very instance, a serous effusion from pressure is an every-day occurrence. However, our author does not deny that pressure on a vein may produce an effect of this kind; but he thus accounts for it:—

"A pressure made on the brachial vein and its branches by schirrous glands in the axilla, is a common cause of this state. The remote cause is here, indeed, of a mechanical kind; but not so the proximate cause of the effusion. By the resistance given, in this case, to the blood's return by the principal veins of the limb, a reaction is occasioned in the extremities of the arteries leading into the corresponding extreme branches of the veins; and which reaction is in this, as in a multitude of other occasions of congestive fulness in these vessels, a sanative effort of nature to overcome the primary obstruction." (P. 111.)

Dr. Ayre next contends against the commonly-received opinion of an anasarcous state of the limbs, in chronic disease, being the product of local and general debility, and which has been supported by observing that the swelling is increased by a depending, and relieved by an horizontal position; by the inflammatory state of the parts being incompatible with this state of debility; and by the want of preternatural heat on the surface of an œdematous part. His answers to these objections are in substance as follow:—First, that the effect is not correspondent with the assigned cause; since, in many cases where the debility is excessive, there is no œdema,—as, for example, in the last stage of fevers. Secondly, that effusion from position will occur in the strongest person, when unduly subjected to this cause; and lastly, with respect to this and other objections, that there is, in certain fatal chronic diseases, a tendency in the lower limbs to take on a low inflammatory action, often of the erysipelatous kind; and that, therefore, the still lower degree of it proper to anasarca may be well imagined to prevail. With respect to the temperature of the surface, this objection, our author truly remarks, may apply to all forms of œdema; yet some are confessedly the consequence of inflammation.

Our readers may now judge whether Dr. Ayre's answers fully meet the objections he has thus fairly stated: we think they do not, and that this section especially restricts the causes of anasarca too much to his favourite explanation, which, though we believe to be generally true, does not appear to include the œdema of old men, that of the pregnant female, and some other similar conditions, which we cannot help still referring to direct debility. We may be wrong, but we are not convinced, by what the author has urged, that we are so.

This section concludes with a recapitulation of the principal positions and doctrines contained in the whole chapter.

The Mode of Treatment to be adopted in the different dropsical complaints above mentioned, forms the subject of the third Chapter. With respect to the mode of treating Hydrocephalus we have but little to remark, excepting that the doctrines and directions are, generally speaking, highly judicious; though the practice does not materially vary from that pursued by the best-informed practitioners of the day. One passage we feel inclined to extract, because our own experience confirms that of our author upon this point; and it is likewise instructive, as it teaches the young medical man to pay attention to any hints which the history of a case may present, by the adoption of which he may occasionally be enabled to perform a cure in very doubtful and obscure cases.

"To some of my readers, perhaps, it may seem like an adoption of the doctrines of the humoral pathologist, to recommend so inconsiderable a remedy as an issue for so considerable an affection as an incipient turgescence, or impending inflammation, of the brain: but, whatever may be said, (and much may be said upon the question,) the fact of its utility in many such cases is indisputable. As an instance illustrative of this fact, among many that have repeatedly fallen under my observation, I may mention here the case of a man whom, some years ago, I admitted into the hospital for epilepsy, which he had been labouring under during a considerable time. The fits occurred three or four times a-week, and were preceded by that peculiar feeling in his right arm which is termed *aura epileptica*. By an accidental exposure of that arm at one of my visits, I discovered a scar, and, upon inquiry as to its origin, I learnt that it was caused by an extensive sore, which had been discharging during several months, and which had healed up a short time only before he was attacked by the fits. The connexion between his disease and the suspended discharge being apparent, I substituted a seton in the neck for the medicines before in use, and with the result, I need scarcely add, of immediately curing him of his epilepsy." (P. 125, 126.)

The treatment of Hydrothorax and Ascites are detailed under the same head, because, says Dr. Ayre, they resemble each other so closely in the nature of their remote or proximate cause. For our own parts, we do not think this a good reason; for, if their causes are the same, the functions and structure of the parts render precisely the same mode of treatment inapplicable to both; and, therefore, we think this section would have been more clear if the treatment of each had been considered separately, with respect to the adoption of venesection, to the employment of mercury, and the use of purgatives. We quite agree with our author in the preference he gives, in *general*, to local rather than to general bleeding, in the diseases leading to dropsical effusion in the cavities. The following remarks upon this subject appear to us to be judicious:—

"With too many practitioners, it is the practice to employ mercury freely in every case of abdominal dropsy, under the vague notion of there existing some mechanical obstruction in the liver or other viscus, as a cause of it; and under the equally vague notion that mercury, so employed, will remove it. The practice, however, (to speak of it in the mildest terms,) is founded on erroneous views of the pathology of these diseases; and employed, therefore, as it is by some, on all the occasions in which they meet with them, must be frequently very injurious. For, independently of the injury to be inflicted by it, when given freely in some of the forms of liver-disease, there is an effect produced by it on the urine, when given to a person in health, resembling that which arises from the specific excitement of dropsy. Under a salivation, the urine becomes charged with serum. Any condition of the system, therefore, approaching even to the state of salivation, must be injurious, by the tendency it must have to increase that morbid state of the body which is nearest allied to the hydropic one. Hence the mercurial salivation has been numbered amongst the remote causes of dropsy; and the resemblance between the dropsical and mercurial excitement, thus established by the common resemblance of the urine in these states, goes far to prove this connexion; and it is not improbable that the mercurial inflammation, when considerable, may survive its specific cause, and degenerate at length into the purely hydropic state. When, however, mercury is given in minute doses, so that these its specific morbid effects are not produced, it is capable of becoming highly useful, as we shall presently have occasion to notice." (P. 148, 150.)

We have seen a case of ascites produced, as far as we could judge, by the action of mercury, excited and kept up for an immoderate period, in a complaint where certainly no visceral affection previously existed.

The only remaining peculiarity in our author's practice in these forms of dropsy, is the small doses of the squill and digitalis which he is in the habit of employing: of squill somewhat less than one grain, and of powdered digitalis only one-sixth of a grain, given uninterruptedly every three or four hours.

The treatment of Ovarian Dropsy we omit, as containing nothing that calls for observation or comment.

On the subject of Anasarca, we are told that, in treating it, we must advert to its nature and causes. In idiopathic cases, it may be treated solely with a view of getting rid of the effused fluid. Here any kind of remedies will occasionally succeed; and puncturing the œdematous parts, with bandages afterwards applied, will be useful. We have found the acupuncturation needle well adapted to fulfil the above intention. Dr. Ayre is in the habit of applying leeches to œdematous swellings, in which the serous inflammation still subsists, and following their application, at the distance of twelve hours, by evaporating lotions; and he tells us that we are to distinguish the cases where this serous inflammation either exists or is making progress, by the state of the pulse and the serous quality of the urine, rather than by the extent of the swelling. In cases, therefore, where the disease is not seen early, the treatment may be frequently limited to those means which promote the absorption of the water; neither leeching nor bleeding will be required. The following directions are important:

"When the dropsy of the skin is considerable and long protracted, and symptomatic of some visceral disease, as it most commonly is in these cases, and is attended by a serous state of the urine and a general failure of the strength, the cachectical state of the system may be considered as established; and the treatment is then beset with difficulties. For the general means, which are useful in the earlier stages of the disease, and when the vital strength is entire, become injurious in this, by the tendency they have, aided by the effects of the visceral disease, to diminish further the vigour of the system; whilst, at the same time, the treatment, which is suited to support the declining strength, can contribute nothing towards lessening the constitutional and local disease, but will frequently increase that morbidly excited state of the circulation, which, analogous to what occurs in diabetes, will continue and increase under the most decided marks of general constitutional weakness. Pending the continuance of that inflammatory state of the system in which the urine is charged with serum, the debility will be mainly derived from that drain of its nutrient parts which is thus established in the body, assisted by the weakening effects of the organic disease. If blood be drawn, it will be found, in many of these cases, to exhibit the usual signs of inflammation; and the treatment of the tonic kind, when employed to support the strength, will be often found to act unfavourably.

"The plan to be pursued must consist in the use of such means as shall assist the powers of digestion and assimilation, so that, by a highly nourishing but plain diet, the drain from the system may be somewhat counteracted; and, at the same time, the cause of the effusion is to be corrected by the use of local depletion and blistering, and by the temperate employment of those general means which are useful in the less aggravated forms of the disease." (P. 174—176.)

It is only necessary to add, that the diet of patients, in the symptomatic form of dropsy, should be plain; in the idiopathic form, the antiphlogistic system should be rigidly enforced; the clothing moderately warm, and such as will promote the insensible perspiration.

Some observations on the treatment of abdominal and ovarian dropsy by tapping, next present themselves to our notice. In all such cases, this operation is to be looked upon as a necessary evil, and only resorted to as a means of avoiding a greater one. The circumstances calling for this operation are the great distention caused by the effusion, producing a morbid stimulus to the peritoneum, or where so much pain and irritation are produced as to risk the, inducing a similar disease of the chest: whilst the objection to it is, the chance of exciting a higher degree of inflammation. Dr. Ayre believes that, in general, tapping is resorted to too early, and under a condition of visceral disease rendering its success impossible. These objections will readily be understood, on reference to the doctrines of the pathology of the disease, which have already been enlarged upon.

"To determine correctly, therefore, regarding the danger of the operation, in respect to the inflammation that may ensue upon it, a reference must be had to the nature and extent of the hepatic or other disease, and not merely to the intensity or the extent of the serous inflammation and its hydropic effusion; both of which are but secondary.

"In illustration of the importance of referring to these distinctions, I may notice the case of a female patient, of about thirty-five years of age, whom I admitted some years ago into the hospital, labouring under an ascites and general anasarca, to a degree that I never saw exceeded. The disease was of some months' standing, and all the usual means had failed with the practitioner whose care she had been under, and who had been only deterred from tapping by the fear of its danger, as her disease was suspected to have originated from intemperance. There were, however, no decided symptoms of hepatic disease, nor any signs of effusion into the chest; and the disease, although formidable in its appearances, and in the disturbance it gave to the breathing, was not so in reality; and the water, therefore, as a measure of necessity, was drawn off by tapping. In three weeks the anasarcous water was absorbed, and there was no return of the ascites. She left the hospital well; and I heard, several years after, that she had since that time continued altogether free from her disease." (P. 182, 183.)

Our author premises a drastic purgative the day before tapping is to be performed. Six hours after, a few leeches are to be applied to the abdomen, and a small blister on each side of it: these are repeated again as early as the condition of the skin will allow. At the same time, the other remedies previously in use must be continued.

The remaining portion of the volume (about fifty pages) consists of cases and dissections of all forms of dropsy,—not occurring, however, in the practice of the author, but selected, with a view to the illustration of the doctrines inculcated in the work, from the writings of eminent practitioners, both English and foreign. It is obvious that this portion of our author's work is not susceptible of analysis; nor can we afford room to extract any cases, since to abridge them would be to rob them of their value. We can do no more than refer the reader for these particulars to the work itself.

In concluding this analysis, we beg to repeat that we have derived considerable information from the perusal of Dr. Ayre's Researches in Dropsy; although we have ventured occasionally to doubt the accuracy of some of his conclusions; and have thrown out here and there a hint, which he may, perhaps, be inclined to adopt when a second edition of the book is called for. There are some points of doctrine which, we think, he may reconsider with advantage, and many that he may enter into at greater length; but the ground-work of his performance is excellent, and, as we said before, his work cannot fail to have a beneficial effect on practice in this class of diseases.

---

ART. II.—*An Essay on the Application of the Lunar Caustic, in the Cure of certain Wounds and Ulcers.* By JOHN HIGGINBOTTOM, Nottingham, Member of the Royal College of Surgeons of London. —8vo. pp. x. 147. London: Longman and Co. 1826.

THERE is certainly nothing very ambitious in the title of this little Essay, which treats of a common substance as an application to injuries as common. We are not, however, among the number of those who measure the importance of medical writings by their pretensions, or estimate the weight of the argument by the weight of the volume. We have before asserted, and we now repeat, that the zeal for surgery too often ends in a passion for what is new and striking; while the more really important cases, of every-day occurrence, are passed by unheeded, and many a student, who could tie a great vessel with dexterity, would be puzzled to heal an old ulcer. The work before us, however, presents an instance of a surgeon who prefers healing the "old ulcer," and who does not despise the task of mitigating the "little evils of human life."

Mr. Higginbottom has been in the habit of treating various common, but painful and often troublesome, surgical injuries, by means of the lunar caustic locally applied, and has brought his observations together in the shape of an Essay, which contains satisfactory proofs of the efficacy of the practice. The nitrate of silver is known to every surgeon as an application of great utility in allaying the irritability of various forms of ulceration, independently of its action as a powerful escharotic. These properties have rendered it available, in the hands of our author, for various useful purposes; but particularly for healing wounds and ulcers by forming an adherent eschar.

"It appears scarcely necessary to describe the immediate and well-known effects of the application of the lunar caustic to the surface of a wound or ulcer. It may, however, be shortly observed, that the contact of the caustic induces, at first, a white film or eschar, which, when exposed to the air, assumes in a few hours a darker colour, and at a later period becomes black. As the eschar undergoes these changes of colour, it gradually becomes harder, and resembles a bit of sticking plaster. In the course of a few days, according to the size and state of the wound, the eschar becomes corrugated, and begins to separate at its edges, and at length peels off altogether, leaving the surface of the sore underneath, in a healed state.

"In the formation of this eschar, several things require particular attention. The application of the caustic should be made over the whole surface of the sore; and, indeed, no part requires so much attention as the edges. To make a firmer eschar, the caustic should even be applied beyond the edge of the wound, upon the surrounding skin; for the eschar, in drying, is apt to contract a little, and in this manner may leave a space between its edges and that of the adjacent healthy skin.

"At the same time, much attention must be paid to the degree in which the caustic is applied. In cases of recent wounds unattended by inflammation, it may be applied freely; but, when inflammation has come on, too severe an application of the caustic induces vesication of the surrounding skin, and the edges of the eschar may in this manner also be loosened and removed. If every part is touched, a slight application of the caustic is generally sufficient.

"The importance of avoiding all causes which might detach the edges of the eschar, will be apprehended by the following interesting observation, which I have been enabled to deduce from very extensive trials of the caustic: it is that, in every instance in which the eschar remains adherent from the first application, the wound or ulcer over which it is formed invariably heals.

"Not only the cause just mentioned, but every other by which the eschar might be disturbed, must therefore be carefully avoided; and especially, as the eschar begins to separate from the healed edges of the sore, it should be carefully removed by a pair of scissors.

"To the surface of the wound the eschar supplies a complete protection and defence, and allows the healing process to go on underneath uninterruptedly and undisturbed. It renders all applications, such as plasters, totally unnecessary, as well as the repeated dressings to which recourse is usually had in such cases; and it at once removes the soreness necessarily attendant on an ulcerated surface being exposed to the open air. In many cases, too, in which the patients are usually rendered incapable of following their wonted avocations, this mode of treatment saves them from an inconvenience, which is to some of no trifling nature." (P. 3–8.)

As it becomes an important object to prevent the eschar from separating, it may be further secured by applying a portion of goldbeater's skin.

Another application of the caustic is to cases in which much and long-continued pain is to be apprehended from the ordinary methods of cure, as in superficial wounds along the skin and similar situations; in which we are told that "the inflammation which would then have been set up, is entirely prevented by the due formation of the eschar." If, however, there be already considerable inflammation round the injury, the caustic is to be avoided, as tending to increase it.

In recent injuries, and in ulcers nearly on a level with the skin, the eschar is generally adherent; and this, of course, is regarded as the most favourable occurrence: but, under other circumstances, pus or a scab forms under the eschar. When fluid is present, (which may be detected by the centre becoming raised, and yielding to the pressure of a probe,) a slight puncture is to be made, the pus evacuated, and the caustic again applied to the orifice thus made. This little operation is to be repeated as often as fluid is found. At length the eschar becomes adherent, and in due time peels off. Where, however, the eschar does not separate favourably, the existence of a scab underneath is to be suspected; under which circumstances, the whole must be removed by the application of cold poultices for two or three days. By this means the eschar is removed, while the inflammation is allayed. The caustic is then to be applied once more, and protected by the goldbeater's skin.

The degree of pain resulting from this method of treatment, is less than would be expected *a priori.* In small wounds, indeed, it is inconsiderable, and of short duration; in recent injuries it is more acute than in ulcers; but, in every case, it is represented as quickly subsiding, and giving place to more effectual relief than the patient would experience under any other method of treatment. Although, therefore, the mere pain does not constitute any very formidable obstacle to its employment, yet there are other circumstances pointed out by the author, as rendering it improper or inefficient,—such as ulcers too extensive to admit of the formation of a complete eschar; or in such a situation as to prevent the possibility of this remaining undisturbed, (as between the toes ;) the presence of much inflammation, or œdema. The lunar caustic has appeared to the author more efficacious than any other, and he gives a preference to that which has been made for some time before it is used.

In many cases it is obviously impossible to treat wounds by means of an eschar, either adherent or unadherent; yet even here Mr. Higginbottom frequently has recourse to the nitrate of silver as a local remedy, applying over it a cold poultice, "made without lard or oil." The application of the caustic is to be repeated every second or third day, till at length, the inflammation having subsided, an attempt may be made to form an adherent eschar.

Having explained, in a general manner, the modifications to be adopted in the application of the remedy, we now come to more particular illustrations:—

"In cases of recent punctured wounds, the orifice and surrounding skin should be moistened with a drop of water; the caustic should then be applied within the puncture until a little pain be felt, and then over the surrounding skin, and the eschar must be allowed to dry. In this manner it is astonishing how completely the terrible effects of a punctured wound are prevented: the eschar usually remains adherent, and the case requires no further attention.

"At a later period after the accident, when the caustic has been neglected, some degree of inflammation is usually present, the orifice is nearly closed with the swelling, and a little pus or fluid is formed within. A slight pressure will evacuate this fluid: the caustic may then be applied within the puncture, and over the surrounding skin beyond the inflammation, and must be allowed to dry. In this manner we frequently succeed in forming an adherent eschar, and all inflammation subsides. Any slight vesication which may be raised around punctured wounds, is not of the same consequence as when an adherent eschar is wished to be formed over a sore or ulcer One or more small punctures may be made to evacuate the fluid, and the part may be allowed to dry.

"If there is reason to think that an abscess has actually formed under the puncture to any extent, it must be opened freely by a lancet, and treated with caustic and poultice, keeping the poultice moist and cold with water.

"In cases of puncture, where the orifice is healed, and where an erysipelatous inflammation is spreading, attended with swelling, I have applied the caustic freely over and beyond the inflamed parts; and I have had the satisfaction to find that the inflammation has been arrested in its progress, and has shortly subsided.

"This mode of treatment is particularly useful in cases of punctured and lacerated wounds from various instruments, such as needles, nails, hooks, bayonets, saws, &c.; and in the bites of animals, leech-bites, stings of insects, &c. In considerable lacerations, the same objection would exist to this treatment as in large ulcers.

"The dreadful effects of punctures from needles, scratches from bone, or other injuries received in dissection, are totally prevented by this treatment. I have, for the last five years, had frequent opportunities of trying it in these cases, and have the most perfect confidence in its success.

"The advantage of these modes of treating punctured wounds will, however, be best explained and established by a selection of cases, to which I can add particular remarks, as they may be suggested by peculiarities in the cases themselves.

"Case I.—A. B. received a severe punctured wound by a hook of the size of a crow-quill, which pierced into the flesh between the thumb and fore-finger on the outside of the hand: scarcely a drop of blood followed, but there was immediately severe pain and tumefaction. The lunar caustic was applied, without loss of time, deep within the orifice, and around the edge of the wound; and the eschar was left to dry. The smarting pain induced by the caustic was severe for a time, but gradually subsided.

"On the ensuing day, the eschar was adherent, and there was little pain; but there was more swelling than usual after the prompt application of the caustic, owing to the mobility of the part.

"On the third day, the swelling remained as before, and there was a little sense of heat. On the fourth day, the swelling and heat had subsided, and the eschar remained adherent. On the succeeding day, the eschar had been removed by washing the hand, and the puncture was unhealed, but free from pain and irritation. The caustic was re-applied.

"From this time the eschar remained adherent, and at length gradually separated, leaving the part perfectly well.

"It is quite certain that, under any other mode of treatment, this severe puncture would have greatly inflamed, and have proved very painful and troublesome; and it is not improbable but that suppuration and much suffering might have ensued. All this is effectually and almost certainly prevented, if the caustic be applied promptly, as in this case. When time has been lost, the case is very different, as will appear hereafter; but, even in these cases, the caustic proves an invaluable application." (P. 24—31.)

The author entertains an equally favourable opinion with regard to the application of the caustic in wounds received during dissection: he states that, at two successive periods, he was brought into great danger from inoculation during the examination of dead bodies; but that, since the last of these occurrences, (in 1819,) he has completely removed this inconvenience by the prompt and free application of the caustic.

In recent punctures, he employs it as described in speaking of simple punctured wounds; but he states that, when the case has been neglected, a small tumor is usually formed under the skin: this is to be removed, and the caustic to be applied both to the surface of the wound and over the surrounding skin. When the case has advanced still further, and the absorbents have become inflamed, a crucial incision is to be made, the caustic very freely applied, and afterwards a cold poultice and lotion; "the usual constitutional remedies being actively enforced."

An analogous method of treatment is recommended in bruises. We give a case in illustration.

"Mr. Granger, aged thirty-six, was exposed to a severe bruise by a great weight of stones, which had been piled up, falling upon the outside of his leg: he was extricated from this situation with much difficulty. Besides the bruise, the skin was removed from the outside of the leg to the extent of ten or twelve inches in length, and in some parts an inch and half in breadth; and, in the fore part of the ankle, a deep furrow was made by the rough edge of one of the stones. I applied the caustic, in about half an hour after the accident, over the whole surface of the wounds, and protected the eschar by the gold-beater's skin. The patient was directed to keep the leg cool and exposed to the air. He took no medicine.

"On the succeeding day, the leg was a little swelled; but the patient did not complain of any acute pain, but only of a sense of stiffness. An adherent and perfect eschar was found to be formed over the whole extent of the wound. There was no fever.

"On the third day, the swelling had abated. No further remedy. The patient was still enjoined to rest.

"On the fourth day, the swelling was nearly gone. The eschar remained adherent. The patient walks about.

"From this time the patient pursued his avocation of a stone-mason: no further remedy was required; no inconvenience experienced; and the eschar separated in about a month.

"I think it totally impossible to have cured this wound, by any other remedy, in less than a month; during which period the patient must have suffered much pain and fever, and have been quite confined.

"It is also quite certain, I think, that there would have been an extensive slough, from the severity of the bruise. This was doubtless prevented by the application of the caustic." (P. 70—73.)

When ulcers are treated in this way, care must be taken that the surface be not too extensive; that it be not exposed to friction or motion; and that it be not attended with profuse discharge. From this it might be anticipated that the remedy would not be successful in extensive ulcers of the legs; but, in small irritable sores, particularly about the ankles, "the caustic is invaluable." Under such circumstances, the cold poultice and lotion should previously be used for a few days, to remove irritability and inflammation. Numerous cases are given in illustration.

Several "anomalous" cases are next detailed, in which this method of treatment was adopted with advantage: among them we find Whitlow, Inflammation of the Finger, Inflammation of the Knee, and Tinea Capitis. Of these, the only part we shall extract is that relating to inflammatory affections of the knee.

"Servant women (I suspect from much kneeling in scouring stairs, &c.) are subject to a species of inflammation of the knee, which is frequently extremely troublesome.

"In one case, suppuration of the integuments took place in the fore part of the knee, and the patient was obliged to leave her situation, and go to her friends at a distance, although every antiphlogistic means was tried for her relief.

"In two other cases, after the application of twenty leeches, and the administration of an emetic and purgative medicine, I applied the lunar caustic freely over the whole surface of the knee, previously moistened with water. In a few hours the cuticle was raised and vesicated. I evacuated a viscid puriform fluid, and I directed the constant application of the cold poultice and lotion.

"In a few days all inflammation subsided, and the patients remained well.

"These three cases having occurred to me at the same time, and being apparently equally severe, I was enabled to judge of the efficacy of this use of the caustic; and I can strongly recommend it to a future and further trial. Its application causes more pain than a blister, but not so much as to form an obstacle to its employment.

"It may not be unimportant here to suggest the trial of the caustic in other cases of inflammation, in which a more than usually active local remedy is required." (P. 126—128.)

It is but justice to the author to state, that his recommendation of the lunar caustic, although certainly more general than that of most practical writers, is by no means universal, and certain cases are enumerated as being decidedly injured by such treatment.

"The caustic is inapplicable in extensive lacerations, for the same reason that it is so in extensive ulcers.

"I have found the caustic of little use in incised wounds, and should not employ it except in such wounds received in dissection.

"I have failed in my attempts to heal scrofulous sores by the adherent eschar: I would propose the trial with the lunar caustic and poultice.

"In erysipelatous inflammation, where vesicles are formed, the caustic does injury, as in recent burns.

"I have always found that the caustic has done injury in boils, aggravating rather than diminishing the affection.

"*Of Burns.*—The application of the lunar caustic in recent burns or scalds, has always appeared to me to increase the inflammation and vesication, even inducing blisters where there were none before. The caustic must not, therefore, be applied in these cases, until the inflammation has entirely subsided; but, when there remains only a small superficial ulceration, the caustic may be passed lightly over the ulcerated surface, to form an eschar, which is to be defended by the goldbeater's skin; for the affection is then reduced to the state of a common superficial ulcer. An adherent eschar is generally readily formed, and no further application is reqnired. If the ulceration be more extensive and deeper, the lunar caustic may he applied, and the eschar treated exactly as in common ulcers." (P. 132—134.)

---

### DIVISION II.

### FOREIGN.

Art. III.—*A Treatise on the Physical and Medical Treatment of Children.* By William P. Dewees, m.d. Member of the American Philosophical Society of Philadelphia, and of the Philadelphia Medical Society; Lecturer on Midwifery, &c.—8vo. pp. 496. Philadelphia, 1825.

This work is about to be printed in this country; but, as we have been favoured with a copy of it from the author, we are enabled to give our readers a brief notice of its contents, without waiting for that event. If we devote less space to the consideration of it than we did to the "Practice of Midwifery," by the same author, it must be remembered that we consider it calculated only to fill up a blank in American literature, of which we have not to complain.

Upon casting our eye over the table of contents, we involuntarily started at the first words, "Chap. 1, of Marriage," and turned back to the title-page, to be assured that the subject of discussion was the moral and medical management of children. We found, however, our first perusal had been correct, and were half inclined to accuse our author of going a little too far back, like Tristram Shandy, who begins his history with his own begetting. We begin, indeed, not merely *ab ovo,* but settle the most remote preliminaries, by comfortably marrying the mother of our future subject in a respectable manner; for the first chapter is occupied in considering the "period of life" at which women may, with every chance of producing healthy offspring, take unto themselves a husband; and in some other observations relating to the subject of marriage, which we confess to be very true, although, perhaps, they may, by some, be thought a little unnecessary and misplaced.

It would appear, from the Preface, that the physical treatment of children is a subject which has not met with that attention in America which its importance demands. The author, therefore, before he enters upon the consideration of the diseases incidental to early life, treats much at length, and with his usual ability, upon every subject relating to this highly interesting duty. The best authorities upon the subject have been consulted, and the experience of Dr. Dewees, which has been very considerable, has of course been added. We have stated our reasons for not dwelling upon many questions which are discussed in this volume. In this country we have numerous works which treat of the "conduct of the mother during pregnancy,"—"the influence of the imagination,"—"on the food proper for pregnant women," &c. &c. although it has not been thought necessary to include these subjects in Treatises upon the Management of Children. Dr. Dewees will, we are sure, take these remarks in good part. Sincerity is our creed, and whether "Tros Tyrius ve," Englishman or American, we state our opinions freely, but we trust fairly.

The volume is divided into two books: the first treats of the Physical Treatment of Children, and the second of their Diseases. In the first book, we believe, no subject is left unconsidered, the knowledge of which is necessary either to the mother of a family, or to a medical practitioner engaged in the management of children; and in America, where it appears so serious a blank was yet to be filled, it will doubtless be read with considerable interest, and much instruction. We would recommend it also to those in this country, who are novices in the various subjects relating to the general management of infants.

Our readers will form some idea of the objects of the second book, by an observation contained in the preface to it:—"As the improvement of any individual in the treatment of one or more of the complaints of childhood, would scarcely justify the writing of a volume to announce them, they are, for the most part, diffused through the Journals of the day: it follows it must become the office of some one to collect and embody them, that they may not be lost, and, by their loss, society sustain an injury. This office we have undertaken."

That Dr. Dewees is particularly well qualified for the task he has imposed upon himself, is well known. He has numbered five-and-thirty years in the active prosecution of his profession, and the diseases of children have claimed a large share of his attention. It is the fashion to look upon the labours of the compiler as of very easy execution, and as undeserving any considerable portion of praise. He is assuredly, however, a much more useful auxiliary in the cause of science, particularly when age and experience have well stored his "mental magazine," than nine-tenths of those who riot in the reveries of hypothesis, and "spin the thread of fancy," to the confusion of themselves and others. We shall not travel over the consideration of the treatment considered necessary by Dr. Dewees in every disease of children, but shall assume the privilege of selecting only here and there a portion of any part which appears to merit particular distinction.

In the *Jaundice* of new-born children, we are recommended, by most authorities in this country, to give an emetic. Dr. Dewees, with the deference which real ability is always prepared to offer to the opinion of others, advises a contrary plan. "We have (he says,) given a fair trial to the remedy, and the result is decidedly against the practice. Emetics have not only failed to relieve the disease, but have rendered the stomach so irritable as not to receive any other remedy willingly." Dr. Dewees recommends the following plan of treatment:

"When we find symptoms of jaundice,—that is, yellow skin, eyes, and urine, we begin by giving small doses of castor-oil,—that is, a small tea-spoonful every two hours, until it purge freely. If, upon the inspection of the evacuations, we do not find bile in them, we follow up the purging, the next day, by giving calomel in very small doses, until a cathartic effect is produced. This may, and does require sometimes, two or three days' perseverance in the calomel, aided by small doses of soda, supersated by carbonic acid gas, before the bowels are moved; for it must be recollected they are most commonly very torpid. We have said we give calomel in very small doses; the following is our formula:—

℞. Calom. ppt. gr. iij.
Sacch. alb. gr. vj. M. bene, div. in xij.

"One of these to be given every two hours until they operate. They are best exhibited in a small drop of thin molasses, washed down by the solution of soda, in the proportion of two scruples to eight ounces of the carbonated water." (P. 274.)

*Erysipelas* in children is occasionally more speedily destructive than in adults, and much discrepancy of opinion exists as to the treatment. The following observations will be new to many of our readers, in answer to the plans proposed by some English writers:—

"We shall say a few words upon each of these plans, before we detail the one usually pursued by ourselves, and other practitioners in this city. 1. As the system evidently labours under high arterial action in the commencement of almost every case of erysipelas, which decidedly requires medical treatment, the bark must not be thought of, either in the first or second stages of this disease. In either of the two last, it may often be proper, if the suppuration or sloughing be extensive. 2. As regards the topical applications recommended by these gentlemen, the cold saturnine have always appeared to us, if not of hurtful tendency, at least of doubtful efficacy. In the early or first stage of erysipelas, the camphorated spirit we have thought occasionally useful, but never efficacious. 3. The propriety of using ammonia in such immense doses, to a child perhaps within a month old, is extremely doubtful, even if it were possible to give it, (which, by the by, in our opinion, is extremely problematical;) but its usefulness in any quantity may be properly doubted, especially as the disease, in its commencement at least, according to Mr. Burns himself, is attended with fever. Of the good effects of calomel purges, we entertain no doubt; but must say, purging with it, or any other cathartic medicine, seems to be much at variance with bark and volatile alkali.

"It is probable that erysipelas may have a number of counteragents; but there are very few, we believe, yet ascertained. In the time of Ambrose Paré, blisters were employed to interrupt the progress of this inflammation, both as regards the extent of surface over which it might be disposed to travel, as well as the terminations of two of its stages in either suppuration or gangrene. This remedy, however, was either forgotten or laid aside for nearly two centuries, because the *modus operandi* of the application could not be explained. To Dr. Physick do we owe its revival, the importance of which can only be appreciated by those who have witnessed the almost wonder-working operation of this remedy. We have frequently succeeded with it, both in the adult and in the child, and can most safely recommend its application, when the inflammation attacks such parts as can readily be covered with a blister.

"The plaster should be of such a size as will rest with certainty upon the sound skin: if this precaution be not taken, its application will avail but little. When the sound skin is well vesicated, the plaster is to be removed, and the part to be treated as if a blister had been used for any other purpose." (P. 278, 279.)

In a former Number of our Journal, we have stated the practice of the American physicians, of applying strong mercurial ointment to the part affected with erysipelatous inflammation. Dr. Dewees particularly recommends this remedy. He covers the inflamed as well as the sound skin with a coat of it; and, when it is removed or becomes dry, it is renewed by a fresh application. As this ointment, however, is used differently in the several stages of the inflammation, a description of the proposed, and indeed practised, method may be useful to English readers who are not generally acquainted with it.

"1. Where the part is inflamed, but not yet vesicated. When we see the inflammation in this stage, we cause the whole of the reddened part, as well as a portion of the sound skin, to be covered with the ointment; which is to be renewed, when the part is deprived of any portion of it.

"2. Where the part is vesicated, but the vesicles not opened. In this case, we cause the vesicles to be carefully opened, and the ointment applied as just directed for the first condition.

"3. Where the vesicles have opened spontaneously, and the part has become encrusted; but the inflammation is spread to either a considerable or limited extent. In this case, we direct the ointment to be applied only to the surrounding inflamed margin, and on a portion of the sound skin.

"4. Where portions have proceeded to suppurate, yet a part of the surrounding skin is inflamed. Under such circumstances, we open the collections of matter as early as possible, and apply the ointment to the margin, as above directed.

"Such is the efficacy of the mercurial application, that it almost immediately arrests the further progress of the disease; therefore, when practicable, it should be had recourse to early.

"We know but one objection to this powerful counter-agent,—the patient sometimes becomes salivated. This, however, seldom or never happens with young children, who are most obnoxious to the disease for which it is prescribed. In adults, on this account, we sometimes prefer the blister." (P. 280.)

The American physicians, Drs. Little and Dean, each claim the merit of this discovery. Their claims appear so nearly equal, that it is somewhat doubtful to which the honour is to be yielded.

We observe nothing peculiar in the internal treatment which is recommended.

Judging from our own observation, erysipelas in new-born children is rarely so severe a disease as it is considered to be by Dr. Dewees.

We do not, however, agree with Capuron* that "la nature seule" may be safely relied upon. We have seen much local irritation and general disturbance in a few cases.

*Of the Suppression of Urine.*—The following case is worthy of attention:—

"Mrs. —— was delivered of a healthy baby, on the 15th of June, 1822. On the 20th, in the evening, the child showed uneasiness; and, on the 21st, it cried violently, and continued to be much pained until the 25th. A variety of simple remedies were used for the relief of the urine, which had been either very sparingly passed or entirely suppressed, most probably from the 20th; but without success. On the morning of the 25th, at ten o'clock, we found the abdomen very much distended, even to the scrobiculus cordis; the skin was shining, and the superficial veins were very much enlarged. The child had several very sparing stools, of a dark green colour. Two tea-spoonsful of castor-oil were given in the course of the morning. At half-past one o'clock P.M. Dr. Parrish introduced a small flexible catheter, and drew off at one time eighteen ounces and a half of a straw or cider coloured urine. At seven o'clock of the same day, the child appeared perfectly relieved; it slept soundly, and took nourishment freely. Two more tea-spoonsful of castor-oil had been given since the visit at noon, but without moving the bowels; nor did any water pass. As the child was easy, it was permitted to rest without disturbance.

"From this time the water was regularly drawn off by the catheter until the child's death, which happened on the 28th. It had gradually declined from the time of our first visit, and its mouth had become very sore. Permission was not obtained to examine it." (P. 282, 283.)

In our review of Dr. Dewees' "Midwifery," we have stated our opinion of the difficulty that must be experienced in introducing a catheter into the bladder of a child of so tender an age,—particularly of the male sex. In the above case the sex is not mentioned. It would appear, however, from the observations of the author, that the operation of passing the catheter, even a few days after birth, is easy of execution.

In several instances we have known infants pass but very small quantities of water. The use of the warm bath, however, has caused a free flow. The necessity for more certain assistance might be required, and it could only be procured by the use of the catheter.

Dr. Dewees is not of opinion that the *aphthous efflorescence* to which children are frequently liable, passes through the whole course of the intestines,—from the mouth to the anus. The occasional appearance of aphthæ at the latter part, he accounts for from the irritating nature of the stools which pass over it. His opinion is verified by one case which terminated fatally, and in which no trace of aphthæ could be discovered in the stomach or intestines. Dr. Good is of a different opinion,† but names no authority in confirmation of his views.

---

* *Maladies des Enfans*, p. 191. Paris, 1813.
† *Study of Medicine*, vol. ii. p. 391.

In the *Colic* of infants, the author considers "a tea-spoonful of warm sweet-oil a remedy of great value." The good effects of castor-oil in such cases need not be particularly pointed out.

The *purulent Ophthalmia* of new-born children is frequently a disease of considerable severity, and long continuance. In many cases it arises from the imprudence of the nurse, by the careless application of soap or spirits to the eye, in washing the infant. Dr. Dewees is of opinion, however, that this formidable complaint always arises in the infant from some "foreign matter it acquires *in transitu*, such as gonorrhœa or leucorrhœa." From this statement we differ, and can only admit the cause he assigns to be an occasional source of the disease. The mucilage of sassafras is employed by Dr. Dewees as an application to the eyes. We are unacquainted with the preparation, but presume it is nothing more than a watery infusion of the wood. The plan of cure proposed by Dr. Underwood does not escape the censure of our author; and, indeed, it must be confessed that the same objection would apply, with equal propriety, to many other parts of his work on the Diseases of Children. We have reason to hope that it is about to appear, with many alterations and improvements from the hands of a very able practitioner of this metropolis. We must, however, in our turn, object to the treatment proposed by Dr. Dewees in this disease; but we do so with respect for his ability, and deference to his long experience. The use of astringent solutions is deprecated by Dr. Dewees, "until after considerable abatement of the inflammation," or "after the disease is so much weakened as to permit the child to open its eyes in a dark room, when we may safely begin to use some mild weak collyrium with advantage." A weak solution of the acetate of zinc is preferred. At the commencement of the disease, leeches and blisters may be required, and purgatives must be given. We grant the propriety of the last-named remedies; but, from the observations we have made upon very many cases of this disease, we are firmly convinced that the profuse discharge from the eye is most effectually arrested, and the safety of the organ itself (which is frequently for weeks covered with matter,) best protected, by the early use of astringent lotions. In every case we have witnessed, where warm poultices and fomentations had been applied, the discharge was considerably increased, and no progress made towards recovery until remedies of the class we mention were used. It is true, we admit, that we may have come to these conclusions from the short and acute inflammatory stage of the complaint having passed by before our assistance was required. But the same circumstance, we presume, must have occurred in the practice of Dr. Dewees.

The remedy we prefer ourselves is that recommended by Mr. Ware, the "aqua camphorata" of Bates's Dispensatory. We do not venture to begin with this styptic application in a greater quantity than half a drachm to one ounce of water. Mr. Ware at once employed it of double the strength we mention.

*Ulceration of the Mouth* is a frequent and obstinate, although perhaps never a fatal, affection of children. Dr. Dewees recommends the following application:—

"℞. Sulph. Cupri, gr. x.

Pulv. C. Peruv. opt. ℨij.

—— G. Arab. ℨj.

Mel. Commun. ℨij.

Aq. font. ℥iij. M. et f. sol.

The ulcerations are to be touched with this mixture and solution twice a-day, with the point of a camel's-hair pencil. This has always speedily put a stop to the disease." (P. 301.)

When children are cutting their teeth, there is often another ulceration of the mouth. "In this complaint the gums become swoln, very dark coloured, and spongy; they bleed from the slightest force; the child drivels constantly; the breath is extremely offensive, and there is always more or less difficulty in swallowing. The teeth that are cut at the time soon decay, and those which were through before the ulceration commenced become injured. We have rarely found any other treatment necessary than cutting the gums, and having the mouth frequently washed with a pretty strong decoction of bark." (*ib.*)

In one family, we have lately seen four instances of this disease, of very unusual severity. Three of them occurred during the second dentition, and were attended with very considerable constitutional disturbance. We applied solutions of alum to the part, and thought the sulphate of quinine necessary as an internal remedy. This form of complaint has been denominated by Dr. UNDERWOOD *aphtha gangrenosa*. Dr. Dewees objects to the term, and properly observes, that it bears no analogy to aphthæ. It is not unfrequently protracted to a very great length of time. Months may elapse before it is remedied, under every advantage of skilful treatment.

We do not find it necessary to abstract any part of the chapter on *Dentition*. The mischiefs frequently arising from this process are clearly and accurately described, and also the best mode of relieving them. One observation, however, which the author makes is interesting, inasmuch as it affords an additional proof of the diversity between the diseases of different parts of the globe. "We *once* saw a case where violent croupy symptoms would appear whenever a tooth was about to be cut; and these would cease when the gums were scarified, or the tooth would come through." In this country, we are certain that any practitioner, with the extensive opportunities of the author of the book under consideration, would never pass a day without witnessing the occurrence of that which it appears he has but once met with. Our readers will be aware that this subject has recently occupied much attention. We have lately inserted some papers relating to it.

*Cholera Infantum* is a disease almost peculiar to the United States. The affections of the alimentary canal, to which children are so obnoxious in other situations, differ materially from the American endemic. It is described at considerable length, and would not be interesting to English practitioners.

Upon the subject of *Hooping-Cough* we have yet much to learn. No disease continues, in many cases, a more obstinate course, even under the best known mode of treatment. There are many opinions also generally entertained respecting it, which are doubtful, if not positively inaccurate. It is not our duty, however, to attempt an essay upon the subject in the present place.

Dr. Dewees observes, that the disease "is sometimes early attended by the peculiar inspiration which gives it its common name. At other times, a considerable period elapses before this takes place; and, *in some cases, it does not at all happen.*" We give the latter part of the quotation in italics, because we consider it of much importance. To many practitioners the fact is doubtless known; but the majority of physicians certainly deem the occurrence of the peculiar noise in breathing necessary to constitute the disease. But such is not the case. For example, in one family where there are several children, and one or two labouring under hooping-cough, we shall frequently find, after an uncertain period, some of the others who have been exposed to the contagion affected with a disease resembling common catarrh, which in the course of some days attacks by paroxysms: no "hooping," however, takes place. We believe such children are not in future liable to hooping-cough. CULLEN tells us, he has had instances of a disease, "which, though evidently arising from the chin-cough contagion, never put on any other form than that of common catarrh."

Although it might be inferred, from the history of the disease as it is commonly drawn, that the diagnosis can never be difficult, it is frequently impossible to determine, at the commencement of the attack, whether we have hooping-cough or merely some slight catarrhal affection to contend with; and it happens, unfortunately for practitioners, that the public always expect an immediate decision, and sometimes, perhaps, attribute our hesitation in declaring the nature of the disease to our ignorance. A positive decision, however, cannot be given "until the permanency and obstinacy of the affection declare it to be hooping-cough." Dr. Dewees observes, that "this disease is generally more severe with infants, as they cannot expectorate with the same freedom as older children." Upon this point, our experience coincides with that of Dr. WATTS, who observes, in his Essay, that "a healthy child at the breast suffers as little from the disease as at any other age."

A medicine called Coxe's Hive-syrup is highly spoken of by the author. After purging with calomel, and bleeding (if required), it is always prescribed; and, judging from the effects of seneca in the adult, we should think it likely to be serviceable when the symptoms of inflammation had abated. To promote expectoration, it is given, to a child of three or four months old, in the dose of eight drops every hour or two. To produce vomiting, the same quantity may be exhibited every fifteen minutes until the operation is produced. We are particularly instructed, however, that the effect arising from a given dose is uncertain, and that we are consequently to be guided rather by the effects produced, than the quantity administered. The following is the recipe:—

"Take of seneca snake-root bruised, squills dried and bruised, each half a pound; water, eight pounds. Boil together over a slow fire, till the water is half consumed; strain off the liquor, and then add of strained honey four pints. Boil the honey and the strained liquor to six pounds, or to the consistence of a syrup; and, to every pound of the syrup, add sixteen grains of tartar emetic; that is, one grain to every ounce." (P. 413.)

Inflammation and congestion of the lungs are frequent concomitants of hooping-cough; "for the relief of which, blisters are found decidedly advantageous after proper evacuations; or, should it be necessary to draw more blood, let it be done by leeches or cups, from between the shoulders. We are decidedly of opinion that blood cannot be taken by leeches or cups, with any thing like the same advantage, from any other part, where there is a threatened congestion of the lungs; and it sometimes becomes important to follow this up by a blister to the same part." (P. 414.)

It has been urged by SYDENHAM, and some other but minor authorities, that hooping-cough will pass through a determined course, and that we can only relieve the pressure of the immediate symptoms. Dr. Dewees, on the contrary, is of opinion "that its duration may as certainly be shortened as the march of fever;" and he has been entirely confirmed in this opinion by the success he once witnessed "from the exhibition of the tincture of the artificial musk, in a family of five children, who were all labouring under confirmed whooping-cough." When this remedy was prescribed, the disease had existed two weeks; "all inflammatory action was completely subdued; and the children were put upon the use of the artificial musk at the same time. One of the youngest ceased to cough in less than a week, and neither of the others continued as much as a fortnight."

In these cases, the catarrhal symptoms were mild, and it was summer. The truth would appear to lie between these opposing statements. To determine with Sydenham, that hooping-cough must run an unabated course, as a matter of necessity, is to yield too little credit to many remedies we possess. But, to assume that we can, with even tolerable certainty, arrest its progress in the majority of cases, would be contrary to our own experience. The inflammatory symptoms, with which, perhaps, the disease invariably commences in a greater or lesser degree, are certainly under our control; but the habitual and distressing paroxysms of hooping and coughing very frequently run on for a considerable period, unchecked by any remedy we apply.

We may mention, also, a fact we have frequently observed, which is not, perhaps, generally known. It is not uncommon for patients who have been affected with this disease, but who have at length entirely recovered, to remain without the slightest cough for an uncertain period, perhaps several months. The cough then returns with its characteristic hooping, and with all its former severity; and, perhaps, again lasts for many weeks. We believe this circumstance is more common in adults. In one case, in our own family, in which we were particularly interested, a lady suffered very severe relapses of the hooping-cough every winter for several years. The application of an opiate plaster to the chest appeared to be of much service in this instance. Dr. Dewees says, "we have never employed any remedy of equal efficacy with the garlic in substance, to relieve the cough of habit after whooping-cough. We have very often used it; and we have rarely seen it fail. The objections arising from its smell are, however, very strong in the minds of some; so much so, that they cannot be prevailed upon to use it. But children of six or seven years, or even older, can very often be prevailed upon to eat it, and become after a while very much attached to it. A child of six or seven may begin by taking a third of a common-sized clove, morning, noon, and evening; gradually increasing the dose as the system becomes accustomed to its action. One of ten may take half a clove three times a-day, increasing it as it may be necessary; and so on for greater ages." (P. 418.)

In the acute stage of the disease, topical remedies can rarely be useful; but afterwards "external applications may be advantageously resorted to,—such as liniments of an irritating nature, as the volatile or the camphorated, the spirit of turpentine mixed with olive-oil, or the juice of garlic, rubbed along the vertebral column. But, above all, we think we have observed more advantage to result from the use of the tartar-emetic ointment, than from any other external application: this should be applied high up between the shoulders.

"It is well understood how much the action of the lungs is dependent on a nervous influence from the spinal marrow; and it is probably on this principle the efficacy of such embrocations is to be explained. The muscles of the chest, diaphragm, and scapulæ, receive portions of the cervical and dorsal nerves; the accessory nerves of Willis form a part of the par vagum, and assist in giving rise to the cardiac and pulmonic plexus: hence the propriety of applications to the spine; and hence the popular opinion of the utility of a Burgundy-pitch plaster between the shoulders is accounted for from anatomical arrangement." (P. 419.)

The progress and treatment of *Croup* are dwelt upon with an attention demanded by the importance of the subject. The only peculiarity we notice consists in the exhibition of Coxe's hive-syrup, above mentioned, during the first stage, either as an expectorant or as an emetic, if necessary. The warm bath is decidedly disapproved of. In the third, "the congestive stage" of Dr. Dewees, it appears that the Polygala seneca, in very strong decoction, is preferred in America to expel the membrane obstructing the trachea. The following is the formula recommended by Dr. Dewees:—

"Take half an ounce of powdered seneca; pour on it half a pint of boiling water, and let it simmer until nearly half reduced; strain it carefully, and give a tea-spoonful every fifteen or twenty minutes, until it puke. This quantity will answer for a child from one to three years old; for one of greater age, two tea-spoonsful at a time may be given; but we believe the decoction should never be weaker than the above."

The medicine exhibited in this strength is apt to run off by the bowels. To restrain the purgative operation, a few drops of laudanum may be added.

The use of spirits of turpentine is suggested, from its undisputed influence upon the mucous membranes. In one case, Dr. Dewees thought it of service in the third stage. The operation of tracheotomy is disapproved of under any circumstances; and the opinion of Cheyne is referred to, as an additional confirmation of its inutility. If attacked early, and with adequate means, the author thinks "there are few diseases so entirely under the control of medicine." We consider this opinion as too strongly expressed.

We sought in vain for some vermifuge medicine, in the observations of the author upon *Worms*, which might fill the blank in our catalogue of remedies. The pink-root (the Spigilia Marylandica), with which we are well acquainted in this country, is considered a valuable article, particularly to expel the *ascaris lumbricoides*. We have frequently found it efficacious. Its operation, however, is occasionally violent and painful. Dr. Dewees has prescribed it many hundred times, and in only one instance has he witnessed any distressing symptoms from it; the occurrence of which he attributes to the medicine being exhibited in too large doses.

Upon the subject of *Scarlatina*, Dr. Dewees observes, "This is reputed to be a contagious disease. On this point, however, the evidence, to say the least, is equivocal. The facts connected with the spreading of scarlatina seem to be perfectly explicable, on the ground of its being epidemic, and not contagious." (P. 467.) We have no doubt ourselves that scarlatina is contagious.

In the chapter on *Measles*, it is stated that it is not settled "whether the constitution can be made to suffer the rubeolous action a second time: evidence is so entirely contradictory on this point, that it would not be safe to draw a positive conclusion either in favour or against it. One thing we, however, may safely declare, that, if it be taken a second time, it is contrary to the ordinary character of this disease." (P. 471.) Dr. Dewees, we presume, is not aware of the cases published by Dr. Baillie, in which measles occurred twice in the same children. We have seen similar instances, even where the first disease had been attended by the characteristic catarrhal symptoms of the complaint. No mention is made of that very common modification of measles, the *rubeola sine catarrho*. This species of the complaint certainly does not exempt children from future attacks of the more usual form of the disease.

We cannot, indeed, but consider this chapter as too superficial, considering the importance of the disease of which it treats. There are many points of high interest, with which the young practitioner should be well acquainted, that are altogether omitted.

We pass over a few other chapters, which treat of *Nettle-rash, Burns, Whitlow, Discharges from the Vagina of young Children*," &c. &c.; as they contain nothing more than brief sketches of the opinions generally entertained, and acted upon in this country.

We cannot conscientiously state it as our opinion that the fame of Dr. Dewees will be materially increased by the present publication. In the first part, trifles are certainly dwelt upon with unnecessary labour; whilst, in the second, we have to regret that we are favoured with but very superficial sketches of many interesting subjects. Some, indeed, of the highest importance are passed over without any notice. It is true the author professes to treat only of those diseases which are incidental to American children; but we can scarcely suppose that inflammation of the brain and its membranes never occurs in children on the other side of the Atlantic; or that the term of Hydrocephalus, which in this country so incessantly assails our ears, is altogether unknown in America.

As a compilation from the best works upon the Diseases of Children, if we are to include those published in this country, the volume we have just noticed is certainly deficient.

---

### MEDICAL AND PHYSICAL INTELLIGENCE.

